# Event-related potential responses of ex-combatants and war victims differ for bias stimuli

**DOI:** 10.1038/s41598-025-27449-0

**Published:** 2025-12-16

**Authors:** Juan E. Ugarriza, Jose D. Lopez, Jhon Quiza, Nataly Vergara, Natalia Trujillo

**Affiliations:** 1 Research Group in Human Rights, Universidad del Rosario, Bogotá, Colombia; 2 Laboratory of Political Psychology, Polipsylab, Universidad del Rosario, Bogotá, Colombia; 3 Faculty of Engineering, University of Antioquia-UDEA, Medellín, Colombia; 4 Engineering Faculty, Universidad de Medellín, Medellín, Colombia; 5 Mental Health Research Group, National School of Public Health, University of Antioquia-UDEA, Medellín, Colombia; 6 Stempel College of Public Health and Social Work, Florida International University, Miami, FL USA

**Keywords:** Armed conflict, Victims, Bias, EEG, IAT, N400, LPP, Social behaviour, Social neuroscience

## Abstract

Antagonistic social identities are reflected in neurophysiological responses to implicit bias, yet empirical validation in real-life post-conflict contexts remains scarce. This cross-sectional study assessed neurocognitive responses to bias-related stimuli among 76 Colombian participants, including both victims and ex-combatants of the armed conflict. Using an Implicit Association Test combined with EEG recording and cluster-based permutation analysis, we examined group differences in event-related potentials. Results revealed differential activation patterns: a language-related N400 and an emotion-related Late Positive Potential (LPP), both showing distinct latencies and amplitudes between groups. Particularly, victims exhibited stronger LPP modulation and delayed mid-to-late components, suggesting heightened emotional processing and increased cognitive load in evaluating bias-laden content. These findings highlight how lived experiences of victimization—not merely exposure to violence—shape neurophysiological processing of intergroup bias. Our results underscore the relevance of implicit cognitive markers for informing psychosocial interventions in transitional justice and reconciliation settings.

**Supplementary Information:**

The online version contains supplementary material available at 10.1038/s41598-025-27449-0.

## Introduction

In post-conflict societies, the lingering effects of violence extend far beyond physical destruction. One of the most persistent and complex legacies is the psychological and social entrenchment of antagonistic identities among groups formerly engaged in the conflict^1^. In Colombia, over five decades of armed struggle have produced nearly nine million officially recognized victims and approximately 70,000 demobilized combatants (Figures as of 2025 provided by the Colombian Agency for Reintegration ARN in Spanish, and the Colombian Government’s Registry of Victims RUV in Spanish. In this context, peace involves much more than disarmament; it requires a profound reconfiguration of how individuals perceive and relate to one another, especially across lines of victimization and perpetration. Today, many victims and former perpetrators live in close proximity, creating daily challenges for coexistence and reconciliation^2^. This unique social configuration offers an important opportunity to investigate how past experiences of violence and trauma shape present-day neural responses to socially charged stimuli.

While the neuropsychological consequences of war—including trauma, stress, and emotion regulation—have been widely studied^3^, the neurocognitive foundations of social division and intergroup bias in post-conflict settings remain underexplored. Understanding how former victims and ex-combatants implicitly process emotionally and morally charged social information is critical for advancing models of social cognition and developing effective reconciliation strategies.

Event-related potentials (ERPs) offer a powerful tool to investigate implicit attitudes, as they provide high temporal resolution of the brain’s response to socially relevant stimuli^4^. ERP components have been widely used to study intergroup bias in domains such as race^5^, gender^6^, and religion^7^. While Implicit Association Tests (IATs) have proven useful in detecting late-stage behavioral expressions of bias^8^, combining IATs with ERP recording allows researchers to identify neurocognitive dynamics involved in the automatic evaluation of group-related stimuli. IAT-elicited ERPs have been linked to cognitive control processes^9^, particularly through mid-to-late components^10^. For instance, Late Positive Potentials (LPPs) recorded in frontal and central regions have been shown to reflect semantic priming and the evaluative emotional salience of a stimulus^11^.

Previous work by our team has documented deep-seated antagonisms between victims and ex-combatants in Colombia, manifested in both explicit attitudes and implicit behavioral tendencies. In IAT experiments involving victims, former guerrillas, former paramilitaries, and receiving communities, participants who self-identified as victims exhibited the strongest implicit biases against former combatants. In Implicit Association Tests, bias-related scores are calculated by comparing participants’ reaction times to bias-congruent versus bias-incongruent stimuli. These scores were directly correlated with the depth of trauma experienced, such as direct or indirect victimization^12^. However, to date, no ERP studies have examined how these war-affected populations process implicit associations tied to their own conflict-related social categories.

ERP research has consistently shown that bias-related processing is reflected in mid-to-late components, most notably the N400—associated with violations of semantic expectation—and the LPP, linked to emotional salience and evaluative conflict.

The N400 component typically emerges around 400 ms after stimulus onset and is thought to reflect the difficulty of integrating semantically incongruent information^13^. It has been elicited across various modalities—written, visual, and auditory^14^, and is especially sensitive to mismatches between pre-activated expectations and presented stimuli^15^. For instance, incongruent sentence endings modulate N400 amplitudes in a robust and predictable manner^16^. In the context of IATs, N400 effects have been attributed to semantic anomalies or expectancy violations, rather than to basic attentional mechanisms^17^. Specifically, larger N400 amplitudes are expected during semantically incongruent IAT trials, such as those associating out-group categories with positive attributes^6^.

Given the well-documented antagonisms and distinct implicit biases between victims and ex-combatants in Colombia, we propose the following hypothesis:

### H_1_

Participants from both groups (victims and ex-combatants) will exhibit heightened N400 amplitudes during IAT trials that present pairings incongruent with their group-specific implicit biases (e.g., in the case of victims, *ex-combatant* + positive attribute, or *victim* + negative attribute), when compared to semantically congruent IAT trials.

In contrast, mid-to-late positive potentials, particularly the Late Positive Potential (LPP), typically emerge in time windows beginning around 300 ms after stimulus onset and can extend well beyond 600 ms, modulated by the emotional significance and motivational relevance of stimuli. Larger LPP amplitudes are associated with sustained attention and elaborative processing of emotionally salient content^18^. This component has been observed during the processing of pleasant, unpleasant, and neutral stimuli—including words, images, and faces—and is typically maximal at centroparietal electrodes, though it can be observed at more frontal sites^19,20^.

In studies of intergroup bias, LPP modulations have been linked to the processing of emotionally or socially charged out-group stimuli, including stereotype-relevant content. Such responses are thought to reflect greater emotional engagement and evaluative conflict. For instance, in the context of a group bias IAT, compatible stimulus pairings (e.g., in-group + positive attribute, or outgroup + negative attribute) tend to elicit stronger LPP amplitudes^6^.

Considering the emotional intensity and moral salience of the Colombian conflict for both victims and ex-combatants, we propose the following hypothesis:

### H_2_

Participants from both groups will exhibit heightened LPP amplitudes in response to IAT trials involving emotionally congruent pairings—those aligned with their conflict-related identities and biases (e.g., negative attributes associated with the out-group)—when compared to incongruent trials.

To test these hypotheses, we conducted an ERP-informed IAT study with Colombian participants who self-identify as either victims of the armed conflict or as demobilized ex-combatants. Our goal is to uncover the physiological mechanisms associated with conflict-related implicit bias by examining two groups with distinct social affiliations. We anticipate that group-specific ERP modulations will reflect long-term exposure to the Colombian armed conflict.

## Methods

### Participants

Between 2016 and 2017, there were a total of 13 municipalities where the Colombian Reintegration Agency, ACR, implemented official community-focused interventions, covering five major regions previously under some form of guerrilla influence.

We applied a stratified sampling procedure and visited 12 of the municipalities targeted by the ACR program. In one additional town, fieldwork could not be conducted due to security concerns. One-time meetings were scheduled as part of the ACR-sponsored activities, and open invitations were sent out in advance by both ACR contractors and our team. Approximately 300 people, based on ACR estimates of the active program population, were invited to participate. A total of 199 individuals accepted the invitation to take part in the Implicit Association Test (IAT) (The number of participants in each town were the following: Cali (8); Chaparral (7); El Bagre (11); Florencia (11); Jamundí (7); Neiva (17); Pasto (14); Popayán (9); Puerto Asís (18); and San Vicente del Caguán (7), Santo Domingo (46), and Medellin (42). In Santa Rosa, security issues impeded our fieldwork). No exclusion criteria were applied at the survey stage. Ex-combatants were pre-identified by ACR contractors, and survey data was subsequently collected.

We utilized a previously validated 18-item Extreme Experiences Questionnaire^21^ to determine which participants from receiving communities were victims. This survey prompted non-combatant participants to indicate whether they had experienced specific war-related traumatic events. As a result, nine community participants were excluded from the study as non-victims.

While our sampling strategy ensures a geographically representative spread, it does not guarantee that the participants themselves are representative of the active population in the ACR program. Participation by community members, most of whom the ACR assumed to be victims, and ex-combatants was voluntary, and attendance consistency was not ensured. Due to the absence of a formal participant list for the voluntary program, assessing any biases introduced by this process is challenging. Ex-combatants and victims who were unavailable during our surveys are not represented, potentially introducing bias against those who might have participated under different conditions. Additionally, it is reasonable to assume that some individuals participated in the program’s early stages but subsequently dropped out, although the ACR did not have available records of such occurrences.

### Measures

We administered 199 questionnaires primarily focused on gathering fundamental demographic information (e.g., age, gender, education level) and assessments of explicit attitudes toward both victims and ex-combatants. Explicit attitude items have been validated and applied previously to a similar population^22^, and are included in Table S1 of the supplementary material.

#### Implicit association test (IAT) design

We employed a computer-assisted Implicit Association Test (IAT) to assess participants’ implicit attitudes toward two social categories central to the Colombian armed conflict: *ex-combatants* and *victims*. Each category was associated with a specific image (a dropped weapon for ex-combatants and a grieving group for victims), while valence categories (pleasant and unpleasant) were represented by a smiling or sad face, respectively. Prior to the test phase, participants completed two practice blocks to familiarize themselves with the stimuli-response format and to reinforce the intended associations. In the first practice block, participants matched group labels (“ex-combatant” or “victim”) with the corresponding image. In the second practice block, they matched valenced words (e.g., “peace” or “pain”) with the corresponding emotional icon. The full list of pleasant and unpleasant words is included in Table S3 of the supplementary material.

Across the task, participants listened to a total of 152 audio-recorded, single-word stimuli (76 pleasant, 76 unpleasant) and were instructed to categorize each word by pressing a response key associated with either the left or right side of the screen, where the valence-related icons were located. Key to the task, these smiling or sad icons appeared near the group-related images, and their positions alternated across blocks to control for spatial bias. Participants were instructed to respond according to the pre-established associations (e.g., matching the audio word, for instance as “good” or “enemy”, to the image it “should” go with, either smiling or sad face), rather than according to their personal opinions.

Blocks 3, 4, 6, and 7 served as the critical test blocks and contained both compatible and incompatible pairings. We defined bias-compatible trials as those in which unpleasant words were paired with victim-related images and pleasant words with ex-combatant-related images. In contrast, incompatible trials paired pleasant words with victims and unpleasant words with ex-combatants. A final block combined both trial types^23^. Block 5 was a transitional block where the key-response mapping was reversed, allowing for counterbalancing.

Each trial consisted of the presentation of an audio stimulus (duration < 1 s), followed by a response window of up to 3,000 ms. Trials completed within the range of 300–3,000 ms and answered correctly were included in the IAT scoring algorithm. Trials answered incorrectly, too quickly (< 300 ms), or too slowly (> 3,000 ms) were automatically repeated. Immediate feedback was provided after each response: a green check mark for correct answers and a red check mark for incorrect ones. The entire task lasted approximately 15 min per participant. The task procedure is illustrated in Figs. S1 and S2 in the Supplementary Material.

To calculate IAT scores, we used the improved scoring algorithm proposed by Greenwald et al. (2003) (A description of the algorithm used for the IAT score estimation is provided in Table S2 of the supplementary file), which accounts for individual variability in response speed and accuracy. Higher scores reflect a stronger pro-victim implicit bias, while negative scores indicate a stronger pro-ex-combatant implicit bias. Table 1 presents descriptors of both explicit and implicit attitudes.

**Table 1:** Explicit and implicit associations between victims and ex-combatants.

	Attitudes toward victims (average score)	Attitudes toward ex-combatants (average score)	IAT Score*	IAT score for subsample (76)
All subjects (199)	− 0.263 (2.656)	3.15 (2.112)	− 0.020 (0.475)	0.048 (0.527)
Groups				
Victims (91)**	0.285 (2.511)	2.166 (2.035)	0.175 (0.390)	0.230 (0.472)
Ex-combatants (108)	− 0.611 (2.724)	3.761 (1.946)	− 0.110 (0.501)	− 0.239 (0.508)
Gender				
Female (116)	− 0.590 (2.290)	2.672 (2.203)	0.093 (0.473)	0.094 (0.496)
Male (82)	0.142 (3.027)	3.755 (1.843)	− 0.083 (0.461)	− 0.209 (0.522)

Victims’ IAT scores were significantly different from those of ex-paramilitaries (p-value < 0.001, BF = 23.845) and ex-guerrillas (p-value < 0.001, BF = 3.766). Additionally, victims’ scores differed significantly from those of ex-combatants taken together (p-value < 0.001, BF = 24.640).

*The IAT score represents the difference in reaction time between compatible and incompatible trial blocks, divided by the pooled standard deviation (standard deviations shown in parentheses). A positive score indicates a positive implicit attitude toward victims compared to ex-combatants, and a negative score indicates the opposite.

**After applying the Extreme Experiences instrument, nine of these participants were excluded in the later stages of the analysis.

On the second column from the right on Table 1, we report mean IAT scores from victims and ex-combatants, 0.175 (SD = 0.390) and − 0.110 (SD = 0.501) respectively. The positive average score among victims reflects that average reaction times in incompatible trials, where they are instructed to associate negative valence audio-recorded words with a visual cue of ex-combatants, and positive with victims, is smaller than in compatible trials, where they are instructed to associate negative valence words with victims, and positive with ex-combatants. In the case of ex-combatants, the negative average score reflects the opposite.

From this pool, we invited participants to take part in an EEG session to examine the anticipated brain activity elicited during the task. Prior to enrollment, participants were questioned about their medical or psychiatric history. Exclusion criteria encompassed a history of severe mental disorders such as schizophrenia, epilepsy, or severe head trauma. Additionally, we excluded five subjects who reported having been both ex-guerrillas and ex-paramilitaries prior to demobilization.

Our final sub-sample comprised 76 healthy Colombian volunteers (44 men, 73 right-handed) with mean age of 37.56 (SD = 10.78; t-test between groups t= -1.103, =p.274). On average, participants had completed 9.98 years of education (SD = 3.51). They were categorized as either victims (23 subjects; mean age in years = 40.21, SD = 12.83; mean years of education = 10.78 SD = 3.63; 21 women, all right-handed) or ex-combatants (53 subjects; mean age in years = 37.05 SD = 10.16; mean years of education = 9.32 SD = 3.61; 42 men, 50 right-handed). Participants were provided with information regarding the study’s objectives, the confidentiality of the collected data, and the procedures involved in the psychological tests and EEG recordings. None of the participants reported any visual nor auditive impairments.

All methods were carried out in accordance with relevant guidelines and regulations, as stated and approved by Universidad del Rosario’s Research Ethics Committee (Minute DVO005-063-CS048, 8 February 2018, and DVO005-234-CS048, 22 August 2019), following the Declaration of Helsinki’s standards. Signed informed consent was obtained from all subjects involved.

### Equipment and settings

We conducted a comparison of the average ERP amplitudes between victims and ex-combatants for both compatible and incompatible trials. Our focus was primarily on the mid-to-late components, restricting the analysis to ERPs between 300 and 800 ms.

EEG recordings were acquired using a 64-channel Biosemi Activ-2 with a sampling frequency of 2,048 Hz, synchronized with the IAT task. Quick-caps were positioned following the 10–20 system, and impedances were maintained below 10 kΩ.

The preprocessing of EEG recordings was executed using the MNE-Python package^24^. After assigning channel locations, signals underwent high-pass filtering with a cut-off frequency of 0.2 Hz^25^. The processing pipeline (PREP) was employed to identify and eliminate channels with extreme amplitudes, those lacking correlation with any other channel, those displaying unpredictability by other channels, and channels exhibiting unusually high-frequency noise^26^. The removed channels were subsequently interpolated. For offline referencing, we utilized the Reference Standardization Technique (REST) rather than legacy techniques such as mastoids or average referencing^27^. Next, after performing ICA analyses, ocular and muscle components were eliminated automatically. Epochs were segmented between 200 and 2,000 ms surrounding the stimuli. Following baseline removal, the signal was downsampled to 256 Hz. We selected trial epochs from blocks 3 and 4, corresponding to compatible IAT blocks, and 6 and 7, corresponding to incompatible blocks. Epochs underwent correction through Bayesian optimization and cross-validation methods to estimate the optimal peak-to-peak threshold for detecting erroneous epochs. Subsequently, a low-pass filter with a cut-off frequency of 30 Hz was applied, and clean epochs were averaged to obtain evoked signals.

We conducted significance tests across 256 time instants on all 64 electrodes to detect ERP components, thereby minimizing the likelihood of type I errors. We relied on univariate cluster-based permutation analysis to determine whether there were statistically significant differences between groups across time and/or sensors, in response to bias-compatible and bias-incompatible stimuli in the IAT^28^. This method of analysis controls for multiple comparisons and ensures that observed differences are not due to random noise.

First, f-scores are computed for each group (time point and sensor), and only those not exceeding a threshold of 4.09 (corresponding to an uncorrected p-value of 0.05) are ignored. Remaining ones are grouped into clusters. The f-scores within each cluster are summed to generate a cluster-level f-score, representing its statistical “mass.” A null hypothesis distribution is then derived from the most extreme cluster-level f-scores across permutations of the data. The statistical significance (p-value) of each cluster is determined by its rank within this null distribution, ensuring correction for multiple comparisons. Each cluster member is assigned the p-value of the entire cluster and reflects an adjustment for multiple comparisons. The multiple comparison-adjusted p-values of samples not assigned to a cluster are set to one. Finally, clusters with p-values below 0.05 are selected.

We then keep only clusters that included at least two sensors and lasted longer than 50 ms, as true effects are more likely to manifest consistently across multiple time points and adjacent sensors. This criterion served as an additional safeguard against false positives. The analysis was conducted using the *spatio_temporal__cluster_test* method from the MNE-Python (Gramfort) package. To determine spatial adjacency between sensors, we constructed the required adjacency array using the package’s method.

This procedure was repeated separately for compatible trials -blocks of negative valence words associated with victims, and positive with ex-combatants-, and incompatible trials -blocks of negative valence words associated with ex-combatants, and positive with victims- so that we could compare both groups under the two conditions.

We then conducted a mass univariate analysis using a 2 × 2 repeated-measures ANOVA, with a between-subjects factor of actor type (ex-combatant vs. victim) and a within-subjects factor of condition (compatible vs. incompatible). This analysis was implemented using the Eelbrain library, which supports unbalanced designs.

## Results

The preprocessing of EEG data revealed that, on average, ex-combatants had 18.01 bad epochs (SD = 23.87), 4.45 interpolated channels (SD = 2.91), and 6.09 bad ICA components (SD = 4.71). Victims had 11.35 bad epochs (SD = 9.78), 4.87 interpolated channels (SD = 2.80), and 8.30 bad ICA components (SD = 4.39). As some participants exhibited an unusually high number of artifacts, we excluded all individuals whose proportion of artifacts (bad epochs, interpolated channels, or bad ICA components) exceeded 30%. This resulted in 23 victims remaining in the analysis, while the number of ex-combatants was reduced by 12, leaving 41. Following this exclusion, the ex-combatants had, on average, 7.51 bad epochs (SD = 9.82), 4.70 interpolated channels (SD = 3.04), and 6.32 bad ICA components (SD = 4.11).

To test our hypotheses, we first examined the effect of trial condition (compatible vs. incompatible) within each group. For victims, a relevant cluster in the 730–810 ms window over the central-parietal region, particularly around electrodes CP1 and C1, showed a significant effect. In this cluster, the mean amplitude for the compatible block was − 0.067 μV (SD = 1.412), compared to 0.549 μV (SD = 1.481) for the incompatible block. A Bayesian t-test provided strong evidence for more positive amplitudes in the incompatible condition (BF = 33.321), consistent with an LPP-like modulation. No significant clusters were found for the ex-combatant group within the corresponding time windows for this effect.

Consistent with our hypothesis regarding the N400 component, we found evidence of modulation in the victim group. For the N400-like modulation between 300 and 500 ms in the frontal region (Fz, F1, F2), we identified, in compatible trials, the expected average amplitude for victims was − 0.871 μV (SD = 1.065 μV), with a median of -0.709 μV, while the expected average amplitude for ex-combatants was − 0.061 μV (SD = 1.000 μV), with a median of 0.072 μV. These results, however, were not found to be statistically significant in the direct within-group comparison.

**Fig. 1:**
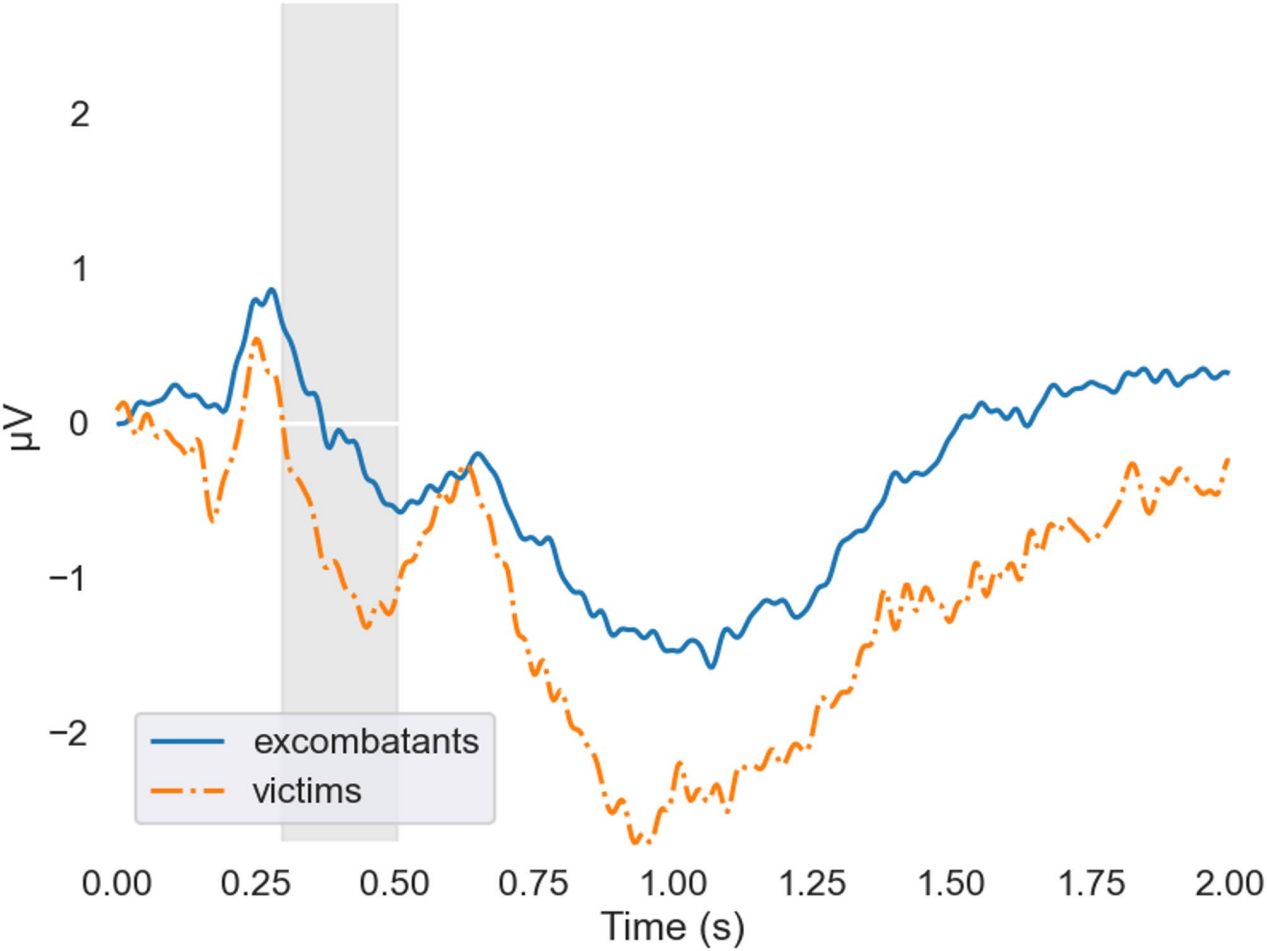
Grand averages of compatible trials in cluster 1. Grand averages of compatible trials for ex-combatants and victims at sensors forming cluster 1. The shaded area indicates the time window of this cluster. Within this window, the grand averages for victims tended to exhibit larger and more negative values compared to those of ex-combatants. Average reaction time for compatible trials is 1,338 ms.

**Fig. 2:**
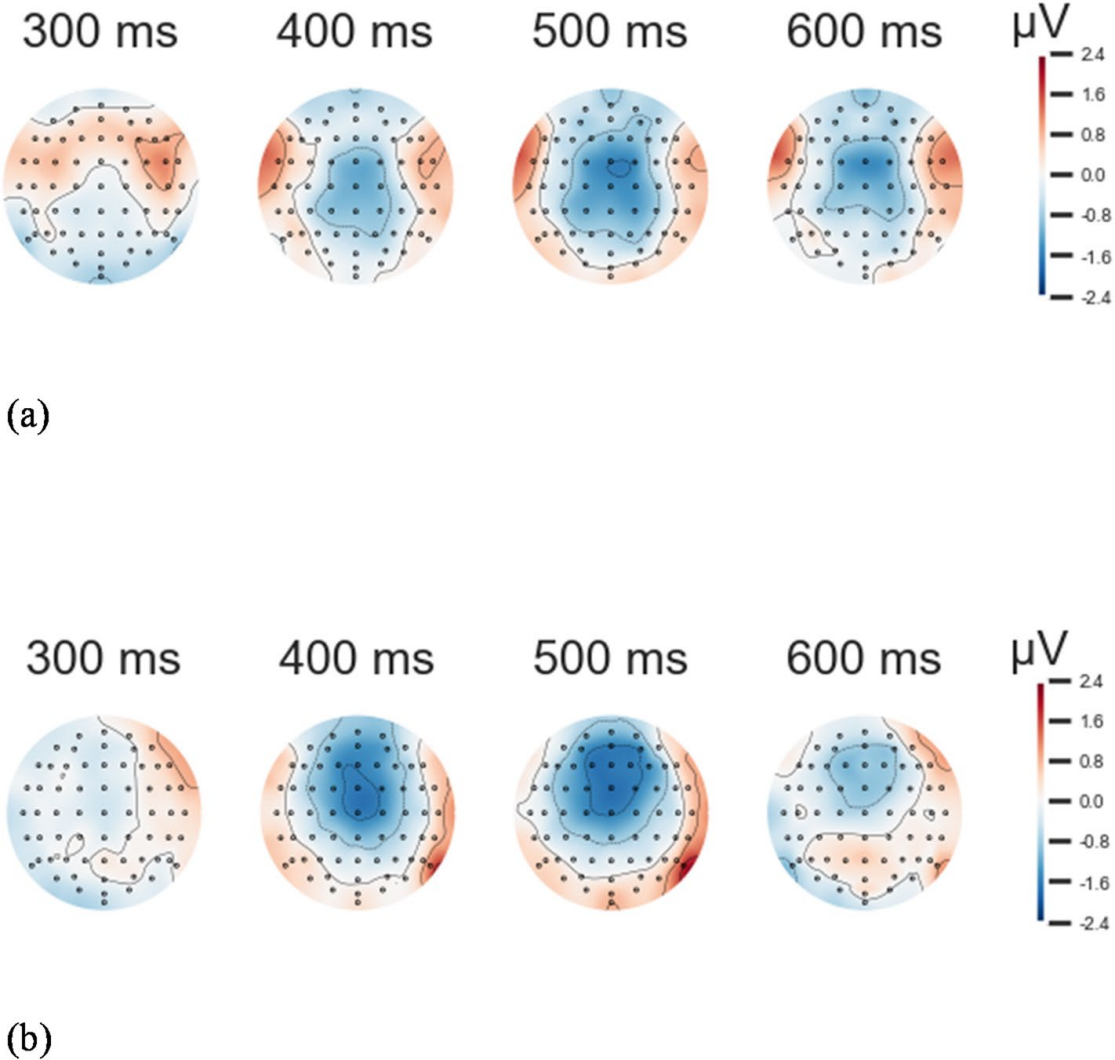
Topographic maps of compatible trials in cluster 1. (**a**) Topographic maps of ex-combatant’s grand averages for compatible trials around cluster 1. (**b**) Topographic maps of victims’ grand averages for compatible trials around cluster 1. In the frontal region, victims’ grand averages tended to show more negative values than those of ex-combatants, particularly between 400 ms and 500 ms.

We also performed a series of Bayesian 2 × 2 repeated-measures ANOVAs on the average amplitudes of the identified clusters to further investigate group-specific effects. For the N400-like component (300–500 ms), the best-fitting model included only the main effect of group, with no evidence supporting the inclusion of a block or interaction term (BF = 3.144). This finding indicates that, in the compatible condition, participants classified as victims showed more negative mean amplitudes than ex-combatants (BF = 21.208). Similarly, in the incompatible condition, victims exhibited more negative mean amplitudes than ex-combatants (BF = 0.753). Grand averages of compatible and incompatible trials are shown in Figs. 1 and 2.

**Fig. 3:**
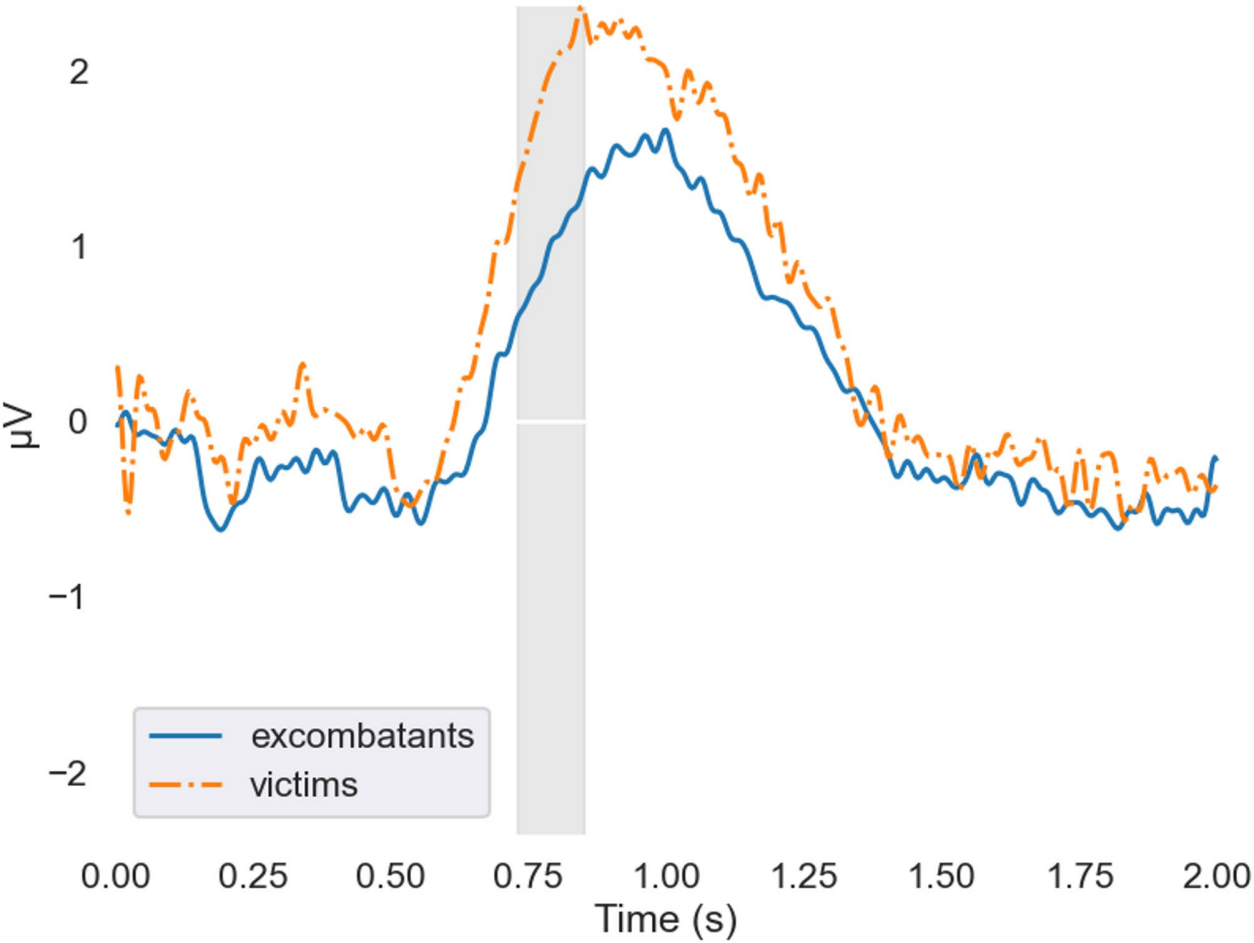
Grand averages of incompatible trials by group around cluster 2. Grand averages of incompatible trials for ex-combatants and victims at sensors forming cluster 2. The shaded area marks the time window of this cluster. Within this window, the grand averages for victims tended to show larger positive values compared to those of ex-combatants. Average reaction time for incompatible trials is 1395 ms.

**Fig. 4:**
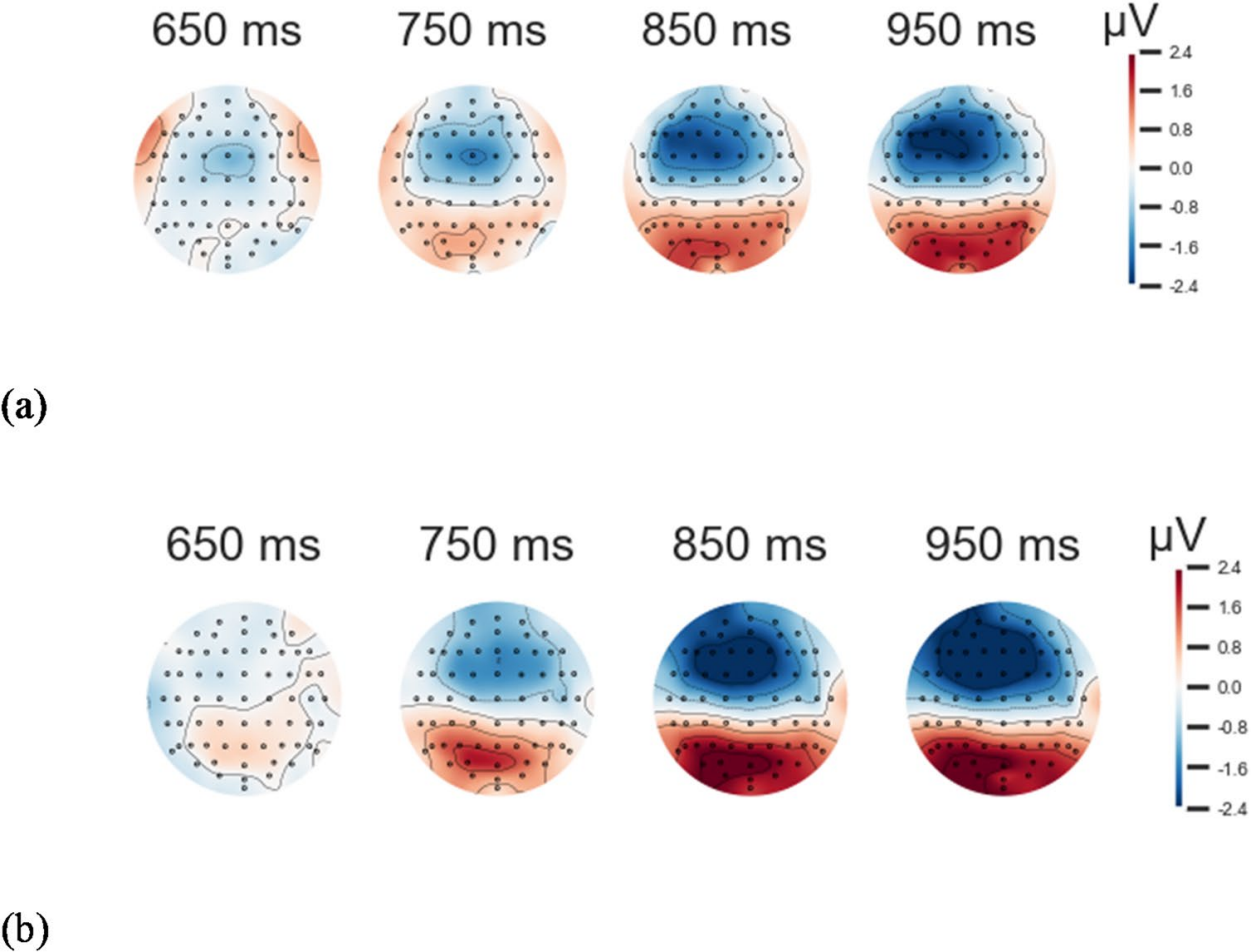
Topographic maps of incompatible trials around cluster 2. (**a**) Topographic map of ex-combatant’s grand averages for incompatible trials around cluster 2. (**b**) The topographic map of victims’ grand averages for incompatible trials around the cluster 2 reveals that, in the parietal region, victims’ grand averages are significantly more positive than those of ex-combatants, particularly between 750 and 850 ms.

For the LPP-like component (730–850 ms), the best-fitting model again included only the group factor (BF = 2.748). This indicates that, in the incompatible condition, victims exhibited more positive mean amplitudes than ex-combatants (BF = 5.843). Likewise, in the compatible condition, victims exhibited more positive mean amplitudes than ex-combatants (BF = 0.701). Grand averages of compatible and incompatible trials for this cluster are shown in Figs. 3 and 4.

Finally, we found no significant differences in these effects between men and women among ex-combatants (N400: *p* = 0.962, df = 39, t = -0.047, d = -0.017; LPP: *p* = 0.314, df = 39, t = 1.019, d = 0.371) or among victims (N400: *p* = 0.797, df = 21, t = 0.261, d = 0.193; LPP: *p* = 0.366, df = 21, t = -0.924, d = -0.683) (Our sample exhibited a notable gender imbalance, with a predominance of men among ex-combatants (42 men, 11 women) and women among victims (21 women, 2 men). This distribution reflects the demographic realities of the post-conflict population, where the majority of victims tend to be women and most ex-combatants are men).

## Discussion

Our study aimed to explore neurophysiological mechanisms underlying war-related intergroup bias using ERP analysis in a real-world post-conflict context. We hypothesized that both ex-combatants and victims would exhibit neural sensitivity to bias-related stimuli, potentially with differing patterns across groups. While prior research has focused primarily on ex-combatants^6^, our findings reveal that victims exhibited stronger mid-to-late ERP modulations, whereas ex-combatants showed attenuated responses.

ERP differences were particularly notable in relation to the type of bias manipulation. We identified two main findings. First, a within-group comparison in the victims’ group revealed that incompatible trials elicited greater LPP-like parietal positivity than compatible ones, potentially reinforcing the idea that bias-congruent associations trigger more emotional or evaluative processing in this population. In contrast, no such differences emerged within the ex-combatants group.

Second, in compatible trials (e.g., positive words with ex-combatants), victims displayed a stronger (more negative) N400-like response than ex-combatants. Although the N400 is classically associated with semantic incongruity, it has also been implicated in the processing of social and normative violations^7^. In our context, this effect may reflect victims’ heightened sensitivity to associations that contradict social identities or lived experience. Consistent with our overall between-group findings, victims also exhibited larger LPP-like responses in a later time window over parietal electrodes. The LPP is generally linked to the processing of emotionally salient or motivationally relevant information^29^. Its enhancement in victims may suggest increased emotional or evaluative engagement with congruent associations involving the outgroup.

Importantly, the observed ERP patterns suggest that victims, more than ex-combatants, engage more intensively with bias-related stimuli.

Our results are consistent with previous studies linking the N400 to violations of social norms and expectations, and the LPP to affective and motivational processes, particularly in trauma-exposed populations^30,31^. However, since the N400 and LPP effects were observed in different trial types (compatible and incompatible, respectively), we refrain from interpreting them as evidence of a unified or sequential processing cascade.

Our finding of a larger LPP for victims in incompatible compared to compatible trials contrasts with our initial hypothesis and with some prior literature on intergroup bias, which has shown larger LPPs for in-group congruent stimuli. This divergence may be a result of the unique and highly emotionally charged context of a post-conflict society. The LPP is a robust index of emotional and motivational significance. In this case, incompatible trials (pairing pleasant words with ex-combatants) may have elicited a stronger LPP because they represent a significant violation of a highly salient lived experience, triggering a deeper emotional or elaborative processing. In a context of war-related trauma, incongruent associations with one’s identity group could be more emotionally salient than associations that are merely congruent with a social stereotype. Therefore, while the direction of our effect is distinct from some previous studies, its magnitude and location remain consistent with the LPP’s established role in reflecting enhanced cognitive and emotional engagement with significant stimuli.

From a broader perspective, these findings suggest that implicit social biases in post-conflict settings are reflected not only at the behavioral or attitudinal level, but also in distinct neurophysiological responses shaped by wartime role. Among victims, greater neural reactivity to biased associations may be modulated by prior trauma, perceived injustice, or identity threat—factors that may remain salient even in the absence of overt hostility^32,33^.

The results partially support our initial hypotheses. First, both groups did show neural sensitivity to bias-related stimuli during the IAT, as reflected in ERP modulations. However, this sensitivity was more pronounced and consistent among victims in the N400 and LPP components, and less so among ex-combatants. These effects suggest that victims may engage in more sustained evaluative and emotional processing when confronted with biased associations—consistent with prior research on trauma and identity threat^31,33^. Nevertheless, the absence of early ERP effects (e.g., N2 or EPN) limits conclusions regarding attentional mechanisms, and points to the need for further investigation of the temporal dynamics of bias processing^34^.

Several limitations should be noted. First, although gender did not significantly influence ERP responses within groups, the gender composition differed across groups, which could introduce uncontrolled variance. This imbalance limits the generalizability of our findings and highlights the importance of gender-balanced designs in future research. Second, the absence of early ERP component analyses constrains our ability to draw conclusions about attentional processing. Third, while ERP amplitudes provide insight into neural engagement, they do not directly reflect specific cognitive operations or the subjective experience of participants. Also, a limitation to our design is the fixed order of the IAT blocks, which was a necessary adaptation for our population and a factor that should be considered in future research. Finally, our exclusion criteria led to the removal of more ex-combatants than victims. Although this was based on objective artifact thresholds, it may have introduced subtle sampling biases that should be addressed in future studies.

In summary, our findings provide preliminary evidence that implicit intergroup biases in post-conflict settings are reflected in neural activity, particularly among victims of violence. These effects appear to be condition-specific and component-specific, rather than supporting a generalized model of biased processing. By combining ERP measures with implicit paradigms, future research could further explore how these neural markers evolve over time, whether they predict intergroup behavior, and whether they can be modified through reconciliation or trauma-informed interventions.

## Conclusion

Our findings suggest that the experience of victimization—more than combat exposure—drives the neurophysiological differences observed between groups, particularly in mid-to-late ERP components. This contributes to a deeper understanding of how bias and prejudice are cognitively processed in conflict-affected populations. However, generalizability is limited by the challenges of field sampling and the specific institutional context of our participants. Future research should adopt continuous measures of conflict exposure to better capture how indirect or civilian experiences of violence may also shape implicit processing at the neural level.

## Supplementary Information

Below is the link to the electronic supplementary material.

Supplementary Material 1

## Data Availability

All data are available in the manuscript or the supplementary materials. Dataset is available at [10.34848/JUCJFP].

## Code availability

Replication code for the cluster permutation analysis is included in the supplementary material accompanying this article.
