# Supplementary material for: Event-related potential responses of ex-combatants and war victims differ for bias stimuli
Source: Sci Rep. 2025 Dec 16;15:43958. doi: 10.1038/s41598-025-27449-0 (PMC12712065; doi:10.1038/s41598-025-27449-0)
Supplement: Supplementary file 1 — Supplementary Material 1 [file 41598_2025_27449_MOESM1_ESM.docx]

|  | *Disagree strongly* | *Disagree* | *Neither agree or disagree* | *Agree* | *Agree strongly* |
| --- | --- | --- | --- | --- | --- |
| (a) Ex-combatants are good for the economy of the country |  |  |  |  |  |
| (b) Ex-combatants rob political spaces to the rest of society |  |  |  |  |  |
| (c) Ex-combatants groups improve the country by bringing new ideas |  |  |  |  |  |
| (d) The Government spends too much on dealing with ex-combatants |  |  |  |  |  |
| (e) Ex-combatants make the country stronger |  |  |  |  |  |
| (f) Ex-combatants increase violence in the country |  |  |  |  |  |
| (g) Victim groups are good for the economy of the country |  |  |  |  |  |
| (h) Victim groups rob political spaces to the rest of society |  |  |  |  |  |
| (i) Victim groups improve the country by bringing new ideas |  |  |  |  |  |
| (j) The Government spends too much on dealing with victim groups |  |  |  |  |  |
| (k) Victim groups make the country stronger |  |  |  |  |  |
| (l) Victim groups increase violence in the country |  |  |  |  |  |
| **Supplementary Table S1. Explicit Attitude Survey Items** | | | | | |

| Step | Improved Greenwald algorithm* | Adapted algorithm |
| --- | --- | --- |
| 1 | Use data from B4 & B7 | Same. |
| 2 | Eliminate trials with latencies les than 10,000 ms; eliminate subjects for whom more than 10% of trials have latency less than 300 ms | Trials whose duration exceeds 3000 ms are discarded. No subject is eliminated. |
| 3 | Use all trials | Same. |
| 4 | No extreme-value treatment | Trials with latencies under 300s are regarded as failed, but they are not discarded. |
| 5 | Compute mean of correct latencies for each block | Sum-up latencies of all trials for each subject to account for task difficulty for marginal populations. |
| 6 | Compute one pooled *SD* for all trials in B3 & B6; another for B4 & B7 | Same. |
| 7 | Replace each error latency with block mean (computed in Step 5) plus 600 ms | No replacement needed due to summation computed in Step 5. |
| 8 | No transformation of resulting values. | Same. |
| 9 | Average the resulting  values for each of the  four blocks. | Same. |
| 10 | Compute two differences:  B6 - B3 and B7 - B4 | Same. |
| 11 | Divide each difference by its associated pooled- trials *SD* from Step 6 | Same. |
| 12 | Average the two quotients from Step 11 | Same |
| * A. G. Greenwald, B. A. Nosek, M. R. Banaji, Understanding and using the implicit association test: I. An improved scoring algorithm. *Journal of Personality and Social Psychology* *85*(2), 197–216 (2003). | | |
| **Supplementary Table S2. Implicit Association Test Score Adapted Algorithm** | | |

**Selection procedures for unpleasant and pleasant words.**

A total of 104 words with either positive or negative intrinsic valence were evaluated by 120 Colombian adults, randomly selected from downtown areas in Bogotá and Medellín. Voluntary participants were asked to rate each word as ‘very pleasant’, ‘pleasant’, ‘unpleasant’, or ‘very unpleasant’. Responses were coded to compute an average valence score for each word, with higher scores indicating greater perceived unpleasantness. The complete list of words is as follows:

*Life, happy, family, free, learn, victory, health, truth, peace, win, opportunity, welfare, caress, fun, friendship, security, effort, friends, party, holiday, calm, value, skilled, care, courage, independent, pacific, Colombia, brave, like, beauty, solidary, cozy, partner, hero, gentle, flower, dear, money, disciplined, nice, compassionate, adaptable, ally, charity, lottery, good, citizen, innocent, rain, country, empathic, fight, cold, winter, married, impulsive, insensitive, lack, stubborn, dislike, scare, bad, tasteless, weak, lose, guilt, worse, dirty, fear, problems, sick, undesirable, danger, poor, anger, horrible, dread, coward, enemy, aggressive, hurt, painful, hungry, disgusting, unemployment, death, jail, gunshot, cruel, falsehood, felony, envy, threat, violence, war, traitor, kill, mistreatment.*

The ten highest- and lowest-rated two-syllable words were selected for use in the Implicit Association Test (IAT) presented in this article. Average valence scores are presented in Table 4.

|  | AVERAGE SCORE |
| --- | --- |
| POSITIVE VALENCE |  |
| Life (vida) | 3.823 |
| Happy (feliz) | 3.747 |
| Free (libre) | 3.722 |
| Health (salud) | 3.689 |
| Win (ganar) | 3.605 |
|  |  |
| NEGATIVE VALENCE |  |
| Jail (cárcel) | 1.462 |
| Horror (horror) | 1.546 |
| Harm (daño) | 1.546 |
| Anger (rabia) | 1.630 |
| Poor (pobre) | 1.655 |
| **Supplementary Table S3. Average valence score for unpleasant and pleasant words.** | |

|  |  |
| --- | --- |
| 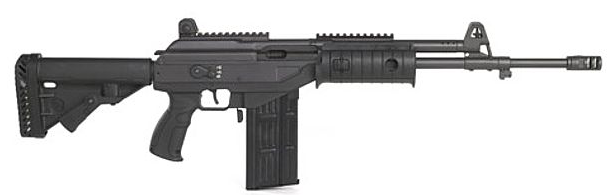 | 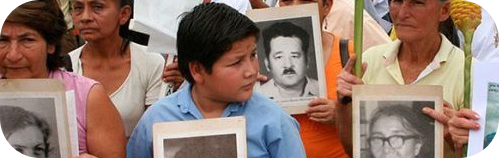 |
| *Associated category: “ex-combatant”* | *Associated category: “victim”* |
|  |  |
| 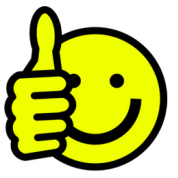 | 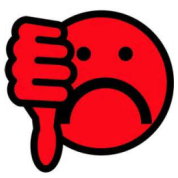 |
| *Associated category: “pleasant”* | *Associated category: “unpleasant”* |
|  |  |
|  | |
| **Supplementary Figure S1. Images and Associated Categories** | |

|  |
| --- |
| 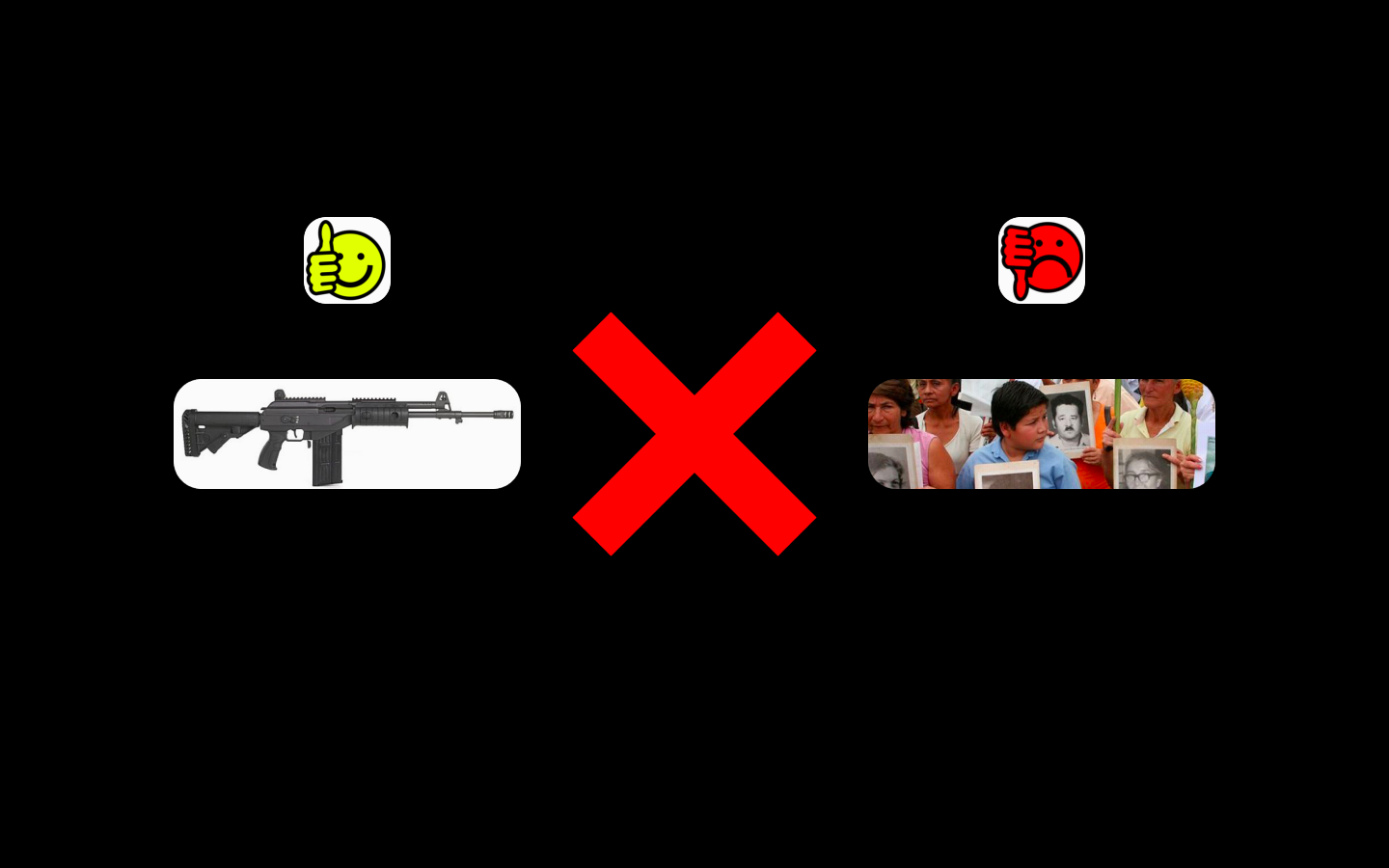 |
|  |
| **Supplementary Figure S2. Example of IAT Test Block Screen** |
